# The microbiome as a modulator of neurological health across the maternal-offspring interface

**DOI:** 10.1172/JCI184314

**Published:** 2025-02-17

**Authors:** Stephanie B. Orchanian, Elaine Y. Hsiao

**Affiliations:** 1 Department of Integrative Biology and Physiology, UCLA, Los Angeles, California, USA.; 2 UCLA Goodman-Luskin Microbiome Center, Division of Digestive Diseases, Department of Medicine, David Geffen School of Medicine, Los Angeles, California, USA.

## Abstract

The maternal microbiome is emerging as an important factor that influences the neurological health of mothers and their children. Recent studies highlight how microbial communities in the maternal gut can shape early-life development in ways that inform long-term health trajectories. Research on the neurodevelopmental effects of maternal microbiomes is expanding our understanding of the microbiome-gut-brain axis to include signaling across the maternal-offspring unit during the perinatal period. In this Review, we synthesize existing literature on how the maternal microbiome modulates brain function and behavior in both mothers and their developing offspring. We present evidence from human and animal studies showing that the maternal microbiome interacts with environmental factors to impact risk for neurodevelopmental abnormalities. We further discuss molecular and cellular mechanisms that facilitate maternal-offspring crosstalk for neuromodulation. Finally, we consider how advancing understanding of these complex interactions could lead to microbiome-based interventions for promoting maternal and offspring health.

## Introduction

Maternal mental health is a cornerstone of public health, impacting the well-being of mothers and the development of future generations. However, few studies to date have focused on the female brain, much less the pregnant or maternal brain. <5% of neuroscience studies from 2010 to 2014 focused solely on female individuals (1). Of all clinical studies from the 1960s to 2013, only 1% were conducted on pregnant women (2). Pregnancy and the postpartum period involve profound physiological and psychological changes that effect the brain. Understanding these changes and the factors that regulate them is critical for supporting women and healthy families.

**Figure 1:**
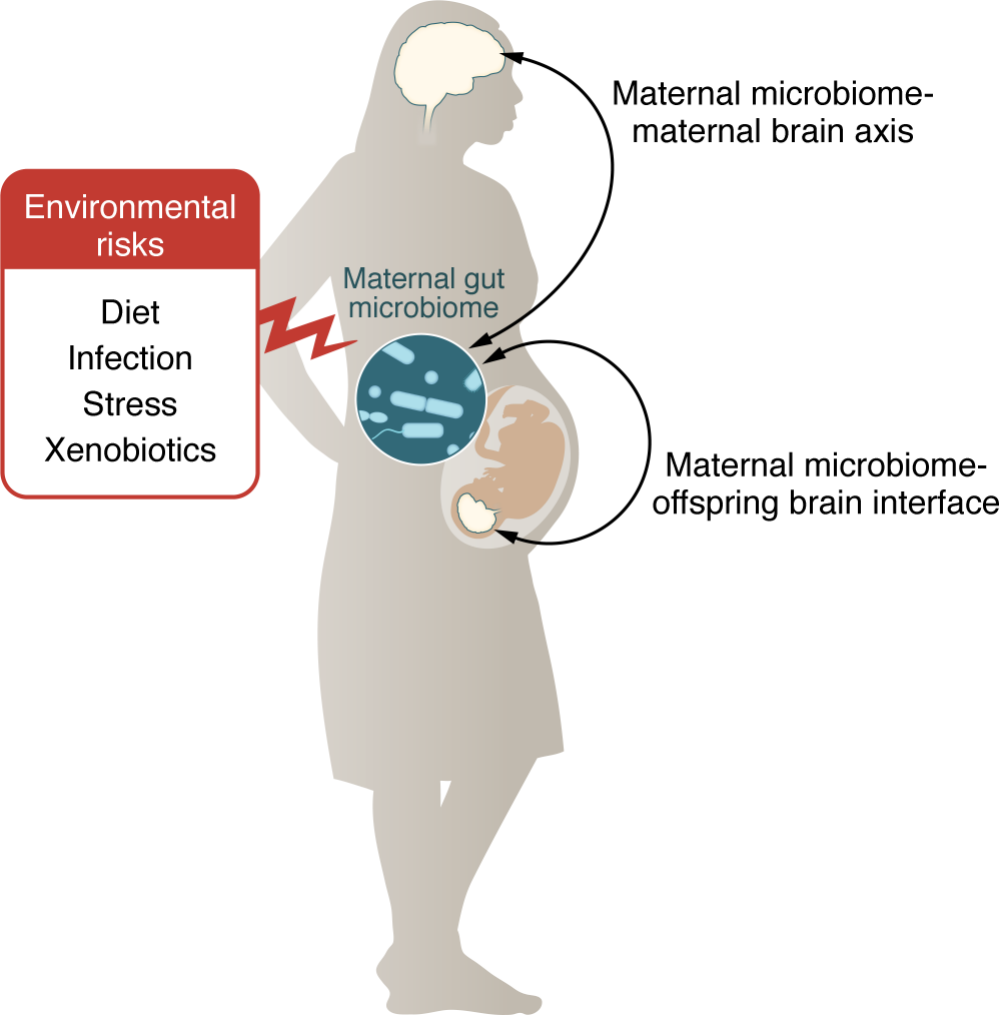
Maternal gut microbiome modulates the maternal and fetal brain. Environmental risk factors, including diet, stress, infection, and xenobiotics, can shape the composition and function of the maternal gut microbiome in ways that impact its interactions with the nervous system in both mother and developing offspring.

The maternal microbiome is emerging as a factor that can influence the health of both mother and offspring. The complex communities of microorganisms in the maternal gut are shaped by perinatal experiences and inform key biological processes of the immune (3, 4), nervous (5), and endocrine systems (6, 7) (Figure 1). Gut microbes differentially respond to sex hormones (8) and exhibit dynamic shifts throughout pregnancy (9). These changes are important, as the maternal microbiome guides physiological processes in the mother and signals to developing offspring in utero through metabolite effectors (10, 11). Furthermore, the transmission of the maternal microbiome from mother to offspring at and after birth informs early postnatal development (12). Given these key interactions between maternal microbiomes and maternal-offspring biology during homeostasis, environmental exposures that disrupt these interactions can lead to increased generational risk for immunological, developmental, and neurobehavioral disorders (13). Herein, we highlight the influences of the maternal gut microbiome on maternal and offspring brain health, with a focus on microbial interactions with environmental factors during the pregnancy period, which together inform the risk for neurological disease. We also discuss growing research on mechanisms for maternal microbial signaling across the maternal-offspring interface.

## The maternal microbiome on maternal brain health

Research on the microbiome-gut-brain axis has gained momentum in recent years, reflecting technological advances (14) and a growing appreciation for the intricate relationships between the gut microbiome and brain health. Gut microbes play a pivotal role in influencing neurological functions and behaviors through various pathways, including the production of metabolites that affect neuroimmune function, neuroendocrine activity, peripheral sensory neuronal signaling, and central neurophysiology (3–5, 15–19). As research continues to uncover the complexities of this bidirectional communication, the need to consider the role of sex differences and gendered experiences on microbiome interactions with the brain becomes increasingly apparent.

Female and male brains exhibit distinct structural and functional differences. Adult male brains had larger volumes, more cerebrospinal fluid, and greater white matter compared with female brains (20). Male brains also showed a greater degree of intrahemispheric communication while female brains exhibit greater interhemispheric communication (21). Additionally, male- and female-specific transcriptomic signatures were found in neurons involved in reproductive behavior and metabolism. During mating and aggression, different subsets of hypothalamic neurons, critical for maintaining homeostasis, were activated in male compared with female individuals (22). These sex-specific reactions to stimuli have also been observed in other regions of the brain, as the amygdala region in female and male brains, important for emotional processing, exhibited differential activity in response to olfactory sensations (23). Additionally, activation of GABA-ergic neurons in the medial amygdala promoted parenting behavior in female mice but infanticidal behavior in male mice (24). Overall, these data describe that neural structures and responses to stimuli can depend on biological sex.

Within females, pregnancy induces neurobiological changes in the brain, reflecting the complex adaptations necessary for supporting fetal development and preparing for motherhood. Hormonal fluctuations lead to structural and functional alterations, including the enhancement of regions associated with maternal behavior, emotional regulation, and social cognition. In mice, pregnancy activated neuronal stem cells and the formation of olfactory interneurons necessary for offspring odor recognition (25). Pregnancy also increased dendritic spines in the hippocampus, which helped to reduce anxiety and promote memory (26). Estradiol and progesterone increased the excitability of galanin-expressing neurons in the medial preoptic area, which promoted parental behavior (27). In humans, reduced gray matter volume in regions linked to social cognition, both during pregnancy and for at least 2 years following childbirth, predicted levels of maternal attachment during the postpartum period (28, 29). Overall, these adaptations illustrate how pregnancy shapes the female brain, optimizing it for the demands of motherhood.

Research is beginning to explore how the gut microbiome can affect brain structure and function between sexes and during pregnancy. Several animal and human studies have characterized sex differences in the gut microbiome (30). These could be driven by direct microbial responses to sex hormones (31–34) and indirect responses to sex-dependent differences in the normal physiology of male and female animals and humans (30). Additional studies emphasize the potential for the gut microbiome to contribute to sex differences. For instance, male but not female germ-free mice had elevated levels of tryptophan and serotonin, and reduced brain-derived neurotrophic factor (BDNF), in the hippocampus (35). Microbial regulation of sex hormones could play a role, as specific microbial species are known to produce enzymes that convert sex hormones into more active or inactive forms, thereby affecting their levels in the body (32, 36). In particular, the microbiome modulated levels of testosterone in mice, increasing it in males and decreasing it in females. Transferring the gut microbiomes from male into female mice conferred elevations in testosterone levels, raising the question of whether these differences would result in alterations in testosterone-related brain and behavioral outcomes and how they may impact pregnancy (37).

While many studies have examined microbiome contributions to brain and behavior, few have focused on maternal mental health and the peripartum period. In pregnant women, particular gut bacteria during the third trimester were associated with anxiety (38). Similarly, in a study of mothers raising young children, reductions in the diversity of the gut microbiome and alterations in levels of particular taxa were associated with high parenting stress (39). While the reproducibility, nature, and directionality of these relationships remain unclear, many are inspired by the promise of microbiome-based interventions for promoting maternal mental health (40–42). Further research is needed to extend and test current principles for microbiome-gut-brain interactions within the context of women’s and maternal brain health.

## The maternal microbiome on offspring neurodevelopment and behavior

### Human maternal infection, antibiotic use, and microbial variation.

Beyond the microbiome-gut-brain axis within individuals, there is increasing appreciation of another microbiome-gut-brain axis that exists between the maternal microbiome and the brain of developing offspring. Interest in this possibility grew from human studies linking maternal antibiotic use in response to infection during pregnancy with neurodevelopmental abnormalities in the offspring. Various types of infections during pregnancy have long been associated with adverse neurological outcomes in children, including mental retardation and developmental delay, psychosis-like experiences, epilepsy, and cognitive deficits (43). Epidemiological and clinical studies examining maternal antibiotic exposure revealed similarly intriguing associations with neurological outcomes in the offspring. For example, a retrospective study of all mothers who gave birth in British Columbia from 2000 to 2014 reported that mothers who filled at least one antibiotic prescription during pregnancy had children with increased risk for autism spectrum disorder (ASD), as compared with those who did not fill an antibiotic prescription (44). In a study of Danish mothers from 1996 to 2004, those who took at least one antibiotic during pregnancy had children with an increased incidence of febrile seizures, as compared with unexposed mothers (45). This was consistent with another study of moms in Korea from 2008 to 2021, wherein antibiotic exposure during pregnancy was associated with a greater risk for epilepsy in children when compared with nonexposed mothers (46). Similar links have been reported for maternal antibiotic exposure and infant attention deficit hyperactive disorder (ADHD), conduct disorder, and mood and anxiety disorders, when evaluating births in Finland from 1996 to 2012 (47). The variety of infections, antibiotics, and maternal gestational periods that have been implicated suggests that there are widespread and generalizable effects of maternal inflammation and antibiotic treatment during pregnancy on adverse neurodevelopmental trajectories in offspring.

Some studies have aimed to correlate maternal microbiomes from healthy women with fetal outcomes to determine whether associations exist in the absence of infection or antibiotic exposure. In mother-child pairs from the United States, particular bacteria from the third-trimester maternal gut microbiome were more strongly associated with child neurodevelopmental outcomes at 1 year of age than was the child gut microbiome (48). Additionally, in a study of mothers from Australia, diversity of the third-trimester gut microbiome predicted their children’s exhibition of internalizing behavior, which is strongly associated with later development of anxiety disorders (49). These studies provide initial evidence that the maternal gut microbiome can correlate with behavioral and developmental characteristics in children, raising the question of whether such relationships may be causal.

### Maternal antibiotic treatment and germ-free rearing in animals.

**Figure 2:**
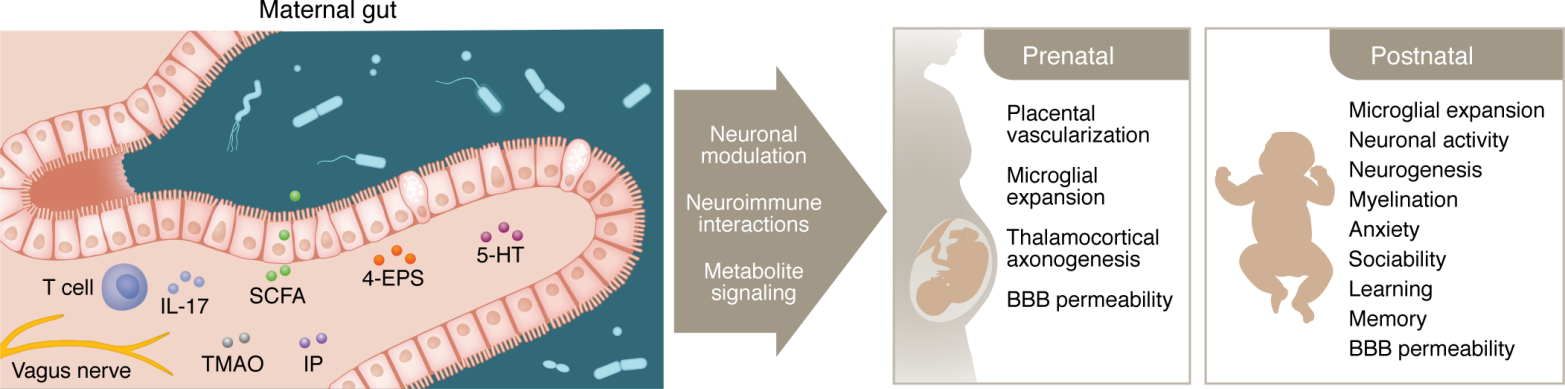
Interactions between the maternal gut microbiome and offspring neurodevelopment. The maternal gut microbiome informs offspring brain and behavioral development through multiple interacting pathways, including the signaling of microbial metabolites to neurons and neuroimmune cells, to modulate peripheral immune responses, peripheral sensory neuronal activity, and central neurodevelopment processes. 5-HT, 5-hydroxytryptamine; 4-EPS, 4-ethylphenylsulfate; SCFA, short-chain fatty acid; TMAO, trimethylamine N-oxide; IP, imidazole propionate.

While many human studies have correlated maternal antibiotic exposure to increased risk for adverse neurological outcomes in offspring, most cases involved antibiotic prescription that was indicated for treating bacterial infection, making it challenging to decouple the effects of antibiotic-induced depletion of gut bacteria from infection-induced inflammation. Animal models studying maternal antibiotic treatment in the absence of infection reveal causal effects of antibiotic exposure during pregnancy on the offspring’s brain and behavioral development. Pregnant mice that were treated with antibiotics yielded fetuses with altered brain transcriptomic profiles and impaired thalamocortical axonogenesis, compared with those reared from vehicle-treated dams (50) (Figure 2). Additional studies indicate that the detrimental effects of maternal antibiotic treatment during pregnancy on offspring neurodevelopment can persist into the postnatal period. Mice treated with antibiotics during pregnancy yielded adolescent offspring with increased blood-brain barrier (BBB) permeability and decreased hippocampal pyramidal neurons, myelination in the corpus callosum, and neurogenesis in the dentate gyrus, as compared with those from vehicle-treated dams (51). In another study, adolescent offspring from dams treated with antibiotics during pregnancy had reduced levels of brain cytokines associated with neuroprotection and repair (52). These findings suggest that the maternal microbiome during pregnancy has a widespread influence, impacting multiple brain regions, cell types, and cellular processes integral to brain development in the offspring.

The lasting effects of maternal antibiotic treatment are further supported by behavioral studies of offspring after birth. In mice, antibiotic treatment during pregnancy and the postpartum period led to anxiety-like behavior and cognitive impairment in offspring, which correlated with reduced expression of N-methyl D-aspartate receptor subtype 2B (NR_2_B), a receptor related to synaptic development, learning, and memory (53). Antibiotic treatment in pregnant mice resulted in decreased locomotion and heightened anxiety in early postnatal offspring. Cross-fostering these pups to untreated dams restored normal behavior, suggesting that adverse effects of maternal microbiome depletion on offspring behavior were reversible (54). Another study found that antibiotic treatment during pregnancy reduced sociability and increased anxiety in the offspring (55). Moreover, adolescent offspring from dams treated with antibiotics during pregnancy showed decreased spatial memory and learning compared with controls (51). While these studies illustrate how maternal treatment with antibiotics, in the absence of infection, can alter offspring neurodevelopment and behavior, they also raise concern that some antibiotics may influence factors beyond the microbiome and elicit off-target effects on the nervous system (56).

As such, many studies examine germ-free animal models, which are devoid of microbial colonization, as a complementary approach to bacterial depletion with antibiotics. When pregnant mice were reared germ free, they produced fetuses with deficient levels of microglia, when compared with controls raised by conventionally colonized dams (57). Fetuses from germ-free dams exhibited reduced thalamocortical axonogenesis, consistent with phenotypes seen from dams treated with antibiotics during pregnancy (50). Additional experiments focused on the transcriptomic pathways affected by deficiencies in the maternal microbiome, finding that fetal brains from offspring of germ-free dams exhibited downregulation of genes involved in neural function and upregulation of genes involved in neuron projection development and glial cell projection (58). They also exhibited distinct metabolic profiles, including alterations in levels of the microbial metabolites 5-aminovalerate (5AV) and trimethylamine N-oxide (TMAO), suggesting that metabolites from the maternal circulation may directly access the fetal brain to alter neurodevelopment.

Alterations observed during fetal development have been similarly reported in germ-free mice at birth and early postnatal ages. Newborn pups from germ-free dams exhibited increased microglia numbers compared with pups from conventionally colonized dams, which correlated with reductions in the cytokines implicated in neuronal differentiation (51, 59). Early postnatal germ-free mice had fewer microglia in the hippocampus and somatosensory cortex and higher cell death in the hypothalamus, abnormalities that were not rescued by cross-fostering to conventionally colonized dams (60). This highlights the importance of the maternal microbiome during the gestational period in programming microglial development in offspring. Similar effects of the microbiome on brain structure have been noted in other species. For example, germ-free zebrafish had fewer neural stem cells and glia compared with conventionally colonized zebrafish (61). Additionally, young germ-free swine had reduced brain weight, along with decreased white matter in the prefrontal cortex and corpus callosum, attributable to decreased oligodendrocyte proliferation (62). These findings underscore the critical role of the maternal and early-life microbiome on offspring neurodevelopment across various animal species.

As germ-free animals exhibit numerous postnatal abnormalities in brain function and behavior, some studies have employed postnatal conventionalization of the microbiome to ask whether restoring the offspring (but not maternal) microbiome can prevent brain and behavioral abnormalities. Restoring the microbiome at weaning failed to prevent elevated hippocampal neurogenesis (63) and serotonin levels (35), hypermyelination in the prefrontal cortex (64), anxiety-like behavior (35), and impaired social preference behavior (65), as seen in germ-free mice. Similarly, early microbial colonization failed to rescue the locomotor hypermotility seen in germ-free zebrafish. These findings highlight the importance of the maternal and/or early-life microbiome in conditioning brain development and behavior in animal models (66). Overall, studies examining the effects of severe microbiome deficiency by germ-free rearing or antibiotic treatment establish proof of concept that the maternal microbiome affects brain development and later life behaviors in offspring. They further raise the question of whether the same principles would apply to more physiologically or clinically relevant contexts.

## Maternal environmental exposures on the microbiome and offspring

### Maternal inflammation.

The microbiome plays a crucial role in regulating susceptibility and response to infection, leading many to consider how the maternal microbiome may modify the effects of infection on environmental risk for neurodevelopmental disorders. Infections during pregnancy have been linked to increased risk for ASD and other neurodevelopmental conditions in the offspring. Maternal fever (67) and infections (68–71) across all trimesters correlate with ASD risk, while maternal exposure to SARS-CoV-2 shows mixed outcomes, with some infants experiencing neurological abnormalities (72, 73) and cognitive delays (74, 75) and others showing no developmental defect. Similarly, in utero exposure to the Zika virus is associated with declines in social communication and motor skills (76–81). Maternal exposures to other infections, such as herpes simplex (82–84) and influenza (85–87), also contribute to adverse child outcomes. The diversity of infections implicated suggests that the generalized maternal inflammatory response during pregnancy drives neurodevelopmental issues. Animal models based on maternal immune activation (MIA), in the absence of overt infection by a pathogen, have established proof of principle that maternal inflammation alters the neurodevelopment and behavior of the offspring. Injection of the viral mimic polyinosinic-polycytidylic acid to activate the immune system in pregnant nonhuman primates and mice altered brain structure in fetuses and early postnatal offspring, including dendritic morphology (88), hippocampal myelination (89), cerebellar development (90), and neuroimmune function (91). These neurodevelopmental alterations corresponded with behavioral abnormalities in adult offspring, including reduced social preference, stereotypies, anxiety-like behavior, and impaired sensorimotor gating, leading many to use MIA to model key features of neurobehavioral disorders like ASD and schizophrenia (91–95).

The microbiome is shaped by the host’s immune status, and immunomodulatory microbes regulate the severity of the inflammatory response. In humans, multiple infections have been correlated with changes in the microbiome (Tables 1 and 2). In mice, dams infected with influenza A virus or *Listeria monocytogenes* during pregnancy exhibited altered gut microbiota by 2 days after infection with risk for long-term “dysbiosis” (96, 97). This was similarly seen in the MIA model, where offspring of immune-activated dams exhibited altered gut microbiota, presumably through vertical transmission of the altered maternal microbiota (98). Treating pregnant dams with antibiotics prevented the abnormalities in social and communicative behavior in the offspring of immune-activated dams, whereas enriching the proinflammatory bacterium segmented filamentous bacteria (SFB) exacerbated abnormal behaviors in the offspring (99). These results suggest that the maternal microbiome can tune the severity of the immune response to maternal challenge to ultimately impact the developmental trajectories of the offspring.

Additional studies have targeted the offspring microbiome to determine whether modifying the microbiome postnatally can impact the presentation of neurobehavioral symptoms resulting from MIA (rather than mitigating the degree of maternal risk itself). Treating offspring of MIA dams with the immunoregulatory *Bacteroides fragilis* at weaning alleviated impairments in communicative, stereotyped, and anxiety-like behaviors (98). Similarly, treating offspring of MIA dams with *Limosilactobacillus reuteri* from birth until weaning improved spatial learning behavior in offspring (100). An additional study tested the provocative question of whether clinical alterations in the microbiome of children with ASD could sufficiently confer brain and behavioral abnormalities upon transfer to mice (101). Colonizing and rearing mice with human ASD-associated gut microbes led to increased repetitive behavior, decreased locomotion, and decreased communication, relative to controls reared with microbiota from individuals acting as healthy controls. Taken together, these studies highlight diverse roles for the microbiome in modulating the severity of maternal risk for immune challenge, as well as the presentation of neurobehavioral symptoms in offspring of immune-activated dams.

### Maternal diet.

Diet is a major determinant of microbiome composition and function, which in turn influences microbial dietary metabolism and nutrient accessibility to the host. Numerous human studies have associated dietary intake during pregnancy with offspring developmental abnormalities. Of these, maternal high-fat diet (HFD) and low-protein diet (LPD) reflect major types of maternal malnutrition that predispose to adverse metabolic and neurological outcomes in the offspring. In particular, maternal HFD was associated with an increased incidence of cognitive impairment and neurodevelopmental and neuropsychiatric disorders, including ASD and ADHD, in their children (102, 103). Similar outcomes have been linked to maternal LPD, even with offspring nutritional habilitation, supporting the importance of the pregnancy period in programming long-term neurological trajectories in the offspring (104). Notably, the similarities in adverse offspring outcomes from maternal diets reflecting overnutrition (HFD) and undernutrition (LPD) highlight the potential for shared pathophysiological pathways.

Animal studies support a causal role for maternal LPD and HFD in disrupting neurodevelopment and behavior in the offspring. Dams fed LPD throughout pregnancy yielded fetuses with decreased neuronal proliferation and increased apoptosis in the ganglionic eminence (105). Upon switching to a control diet at parturition, offspring of LPD-fed dams still developed impaired cognitive behavior and increased anxiety-like behavior by adulthood, highlighting the importance of maternal diet during the pregnancy period (106). In contrast, dams fed HFD before and during pregnancy yielded fetuses with altered neuronal proliferation in the hippocampus and cortex (107, 108). Continuation of HFD through the lactation period led to anxiety-like behavior (109–112) and reduced sociability in the offspring (113). Although maternal diets can affect fetal development through mechanisms independent of the microbiome, alterations in the maternal microbiome have been reported in animal models of maternal LPD and HFD. LPD reduced the diversity of the maternal microbiome during pregnancy, with notable shifts in several *Clostridial* species and corresponding microbiome-dependent alterations in metabolomic profiles across the maternal-fetal compartments, including in the fetal brain (106). HFD in pregnant mice induced differential temporal shifts in the maternal microbiome, with early enrichment of *Akkermansia* and *Bifidobacterium* and later alterations in multiple *Clostridial* taxa (114). Different microbial signatures were reported in nonhuman primates fed HFD, highlighting the potential for host species-specific effects (115).

Alterations in the maternal microbiome are also associated with dietary intake in pregnant women (Tables 1 and 2). Only a few studies to date have evaluated causal roles of the microbiome in modifying the effects of altered maternal diets on neurobehavioral outcomes in the offspring. In a study of maternal LPD, which reduced the diversity of the maternal microbiome during pregnancy, further depletion of the maternal microbiome via antibiotic treatment exacerbated cognitive and anxiety-like deficits in adult offspring that were reared on a control diet since birth. In contrast, maternal supplementation with select microbial metabolites during pregnancy partially prevented abnormal behaviors in the offspring.

Microbiome manipulations in models of maternal HFD have as yet focused on postnatal interventions to mitigate social impairments in offspring (106). Normalizing the microbiome by cohousing offspring from dams fed HFD versus control diet, or by transferring fecal microbiota, ameliorated the social behavioral deficits caused by maternal HFD. Positive effects were similarly seen by treating HFD offspring with *Lactobacillus reuteri* for 4 weeks after weaning, which corresponded with increases in oxytocin-reactive neurons in the hypothalamus (116). As with MIA, these studies highlight the ability of the maternal microbiome to modulate the severity of maternal malnutrition on promoting abnormal behavior in the offspring as well as the ability of postnatal manipulations of the offspring microbiome to modify behavioral symptoms arising from maternal insults.

### Maternal stress.

Microbial “dysbiosis” has long been associated with exposure to stressful situations (117) and altered stress response (18), raising interest in potential relationships between the maternal microbiome and perinatal mood disorders. Maternal anxiety and depression are linked to many negative outcomes in child neurodevelopment, including reduced cognitive and social-emotional performance (118, 119) and increased risk for emotional disorders and ADHD (120–122). Dams exposed to periconceptual stress yielded fetuses with alterations in brain gene expression (123–125), including in pathways related to neuronal development, core metabolism, and neuroimmune function, with notable sex differences. Consistent with this, many studies have reported effects of maternal stress on offspring neurogenesis (126–128), tryptophan and amino acid metabolism (129), microglial and cytokine levels (130–132), as well as anxiety-like behavior and impaired cognitive behavior (127, 133).

Early culture-based observations that stressful environmental and housing conditions correspond with rapid decreases in *Lactobacillus* (117) have generally aligned well with more modern sequencing-based studies of stress-induced alterations in the gut microbiome (134, 135). Recent studies have extended this line of inquiry to the maternal microbiome, finding that maternal stress alters microbial diversity, with particular increases in *Lachnospiraceae* and *Oscillibacter* and decreases in *Parasutterella* (136). Similar observations of stress altering the maternal microbiome have been observed in humans (Tables 1 and 2). Studies also report the ability of maternal stress during pregnancy to alter the offspring microbiome, with decreases in *Lactobacillus* and *Ruminococcaceae* (129) and increases in *Prevotellaceae* (128, 137). One particular study manipulated the maternal microbiome to alter maternal, rather than offspring, neurological health and found that treating stressed dams with *Lactocaseibacillus rhamnosus* HN001 during pregnancy reduced their anxiety-like behavior, with corresponding alterations in cortical neurotransmitter levels (138). These studies raise the prospect of modifying the maternal microbiome to offset the adverse effects of maternal stress during pregnancy on both maternal and offspring neurological health.

### Maternal antidepressants.

While most studies of the maternal microbiome have interrogated its ability to modify *risk* for neurological disorders, there is rising interest in the roles for the maternal microbiome in regulating *responsiveness* *to treatments* for neurological disorders. As an extension of interest in maternal depression and anxiety, the microbiome is increasingly implicated in interacting with common antidepressant and anxiolytic drugs, including selective serotonin reuptake inhibitors (SSRIs) and serotonin-norepinephrine reuptake inhibitors (SNRIs), for which maternal use during pregnancy has been associated with increased risk for ASD and ADHD in offspring (139–146). In large-scale screens of microbiome interactions with medications, SSRIs exhibit notable associations with microbiome alterations and effects on microbial activity (147–149). These links have been further assessed in animal models, wherein maternal SSRI treatment altered the composition of the maternal gut microbiome. In dams deficient in serotonin transporter (SERT), a model for maternal depression, treatment with the SSRI fluoxetine throughout pregnancy reduced *Bacteroides* and increased *Prevotella* and *Ruminococcus* levels in the gut (150). In contrast, treating wild-type dams with fluoxetine from midgestation through lactation increased *Parasutterella* and decreased *Turicibacter* (151). Another study that treated wild-type mice with fluoxetine during midgestation reported selective increases of *Lachnospiraceae* COE1, a short-chain fatty acid–producing (SCFA-producing) bacterium (152). To gain insight into whether the maternal microbiome may modify the effects of SSRIs on the host, the study examined maternal fluoxetine treatment during midgestation in microbiome-deficient dams compared with conventionally colonized dams (152). Maternal fluoxetine treatment resulted in gene expression alterations in the fetal brain, including in genes related to synapse organization, cognition, locomotory behavior, and neurotransmission. These signatures were altered by maternal treatment with antibiotics to deplete the microbiome prior to fluoxetine exposure, establishing proof of principle that the presence of the maternal microbiome modifies the effects of maternal fluoxetine exposure on offspring neurodevelopment. Taken together, results from these studies emphasize that the maternal microbiome is an important factor that can modify the severity of maternal environmental exposures and their effects on both maternal and offspring health.

## Mechanisms for microbiome-brain interaction across the maternal-offspring interface

### Microbial effects on maternal-offspring barriers.

The ability of the maternal microbiome to modulate offspring behavior has motivated many to uncover signaling pathways that mediate crosstalk between maternal gut microbes and the developing brain. As the literal interface between mother and fetus, the placenta plays a critical role in supporting fetal neurodevelopment by ensuring the proper supply of nutrients, oxygen, and hormones and protection from pathogens and harmful substances (153, 154). In mouse models of maternal stress and MIA, placental inflammatory responses were quickly mounted in response to the maternal challenge and required for downstream neurological abnormalities in the offspring (155–157). Similar relationships have been reported in large epidemiological studies, wherein small placental size and placental pathologies were associated with ADHD, antisocial disorder (158), and poor early learning in children (159).

Recent studies have asked whether the maternal microbiome impacts placental physiology and function. Pregnant mice reared germ free or treated with antibiotics yielded placentas with reduced weight, volume, and tissue density, compared with conventionally colonized controls (160). These morphological deficits were localized to the placental labyrinth region as the main site for maternal-fetal exchange and corresponded with deficient placental vascularization. Consistent with this, placental metabolomic profiles from germ-free dams were altered compared with those from conventionally colonized controls (161). In particular, maternal supplementation with SCFAs, products of bacterial fermentation by the gut microbiome, restored placental weight and vascular development in microbiome-deficient dams (160). Consistent with the ability of SCFAs to cross the placental barrier, the effects of SCFA on placental vascularization were mediated by direct signaling to the G protein–coupled receptors GPR41 and GPR43 on endothelial cells (162, 163). Upon entry into the fetus, receptor-mediated signaling of SCFAs regulated fetal inflammation (164), lipid metabolism (165, 166), and insulin secretion (167, 168). These studies indicate that the maternal microbiome can impact fetal health by indirectly modulating placental function and producing metabolites that cross the placental barrier to signal to fetal tissues. While there is a strong premise for placental influences on fetal neurodevelopment, how microbiome-dependent regulation of placental function may ultimately impact brain health of the offspring remains to be studied.

The gut microbiome regulates hundreds of bioactive metabolites across various organ systems, raising the question of whether this ability may extend from the maternal microbiome to metabolites in the fetus. Indeed, serum metabolomic profiles from fetuses of microbiome-deficient dams were altered compared with those from conventional controls (50, 160). Given that subsets of metabolites from the maternal microbiome, like SCFAs, can enter the fetus directly by crossing the placental barrier, a key question is whether they may similarly access the offspring’s brain by crossing the BBB. The BBB is not fully developed until after birth (169), suggesting that microbial metabolites that enter the fetus have the potential to access the fetal brain. Indeed, several metabolites in the fetal brain were differentially regulated by maternal microbiome status, and a subset of these were coregulated in maternal serum (50). There is also some evidence that the microbiome may modulate BBB development. In germ-free mice, the BBB remained permeable even after birth, with decreased expression of tight junction proteins, compared with conventionally colonized controls (170). Restoring the microbiome or supplementing with SCFAs after birth rescued these phenotypes (171). This relationship between the microbiome and BBB was similarly seen in other disease-related models. For example, offspring of immune-activated dams exhibited increased BBB permeability compared with those from vehicle-treated controls (172), which was corrected by maternal treatment with *L*. *reuteri* during lactation (100). Overall, these results indicate that the microbiome influences BBB integrity and that the maternal microbiome modulates metabolites that access the developing brain in offspring.

### Signaling of microbial metabolites to neurons.

Given that the microbiome modulates numerous metabolites within the developing brain, recent studies have asked whether the maternal microbiome alters offspring neurodevelopment via the regulation of metabolites that act upon neurons. By screening metabolites on fetal brain explants cultured ex vivo, microbially regulated metabolites, including TMAO and imidazole propionate, were found to promote thalamic axon outgrowth. This was further validated by supplementing microbiome-deficient dams with the microbial metabolites of interest, which prevented defects in thalamocortical axonogenesis and later-life tactile sensory behavior in adult offspring (50). Similar influences of metabolites on offspring behavior were seen in a mouse model of maternal protein undernutrition, where supplementing protein-restricted dams with a cocktail of 10 microbially modulated metabolites prevented cognitive deficits and anxiety-like behavior in adult mice that were reared on a standard diet since birth (106). These studies indicate that metabolites regulated by the maternal microbiome have the capacity to act directly on the fetal brain to impact the neurobehavioral development of the offspring.

Other evidence of microbial metabolite interactions with neurons is derived from postnatal studies. Many brain metabolites are modulated by the microbiome during adulthood, opening the possibility that they may regulate the activity (rather than development) of neural circuits underlying behavior. In mice colonized with gut microbes from patients with ASD compared with mice colonized with gut microbes from individuals acting as healthy controls, decreases in brain levels of the 5AV and taurine were associated with deficient social and stereotypic behaviors. Causal links were established, wherein supplementation of the metabolites reduced repetitive behavior and increased social duration. The metabolites were proposed to directly modulate neuronal activity, as 5AV reduced the excitability of pyramidal neurons, and taurine delayed the switch from excitatory to inhibitory response to GABA in cortical neurons (101). Additional microbial metabolites were reported to indirectly modulate neuronal activity by regulating neuronal myelination in the brain. The microbiome-dependent metabolite 4-ethylphenylsulfate was abnormally elevated in mouse models of ASD and reduced oligodendrocyte maturation to promote anxiety-like behavior in mice (173). Whether these effects may extend to early neurodevelopment of oligodendrocytes, during midgestation in the mouse, remains unclear.

In addition to interacting with neurons within the central nervous system, many microbially modulated metabolites signal to peripheral sensory neurons either directly or indirectly via intermediate effectors. Recent studies highlight that microbial metabolites from the intestinal lumen regulate the activity of gut-innervating vagal neurons that project directly to the brain stem (174) and also to gut-innervating dorsal root neurons that signal through spinal circuits to the brain (175). Consistent with this, gut microbes regulate a variety of behaviors in a manner that requires an intact vagus nerve (116, 176). Beyond active sensory signaling of microbial metabolites, there is also evidence that the microbiome may regulate the development of neuronal circuits for interoception. Mice reared germ free exhibited widespread alterations in vagal neuronal gene expression, including reductions in urotensin 2B (UTS2B) — a putative regulator of blood pressure, which may influence its response landscape. The vagus nerve is reported to be functionally active during early gestation (177), raising the intriguing possibility that metabolites regulated by the maternal microbiome may begin to exert their effects on vagal activity during fetal neurodevelopment. Further research is needed to explore mechanisms for metabolite interactions with neurons, especially during fetal and early postnatal critical periods.

### Microbial regulation of neuroimmune function.

The immune system plays an integral role in guiding normal neurodevelopment, through both central functions of brain-resident immune cells and peripheral immune signaling to the brain. Mice reared germ free or treated with antibiotics exhibited deficiencies in brain microglia, characterized by cellular immaturity and dysfunctional response to immune activation (178). Microbiome-dependent alterations in microglia were even seen in prenatal ages (57), suggesting a role for the maternal microbiome in affecting microglial development. However, supplementation of adult germ-free mice with microbial SCFAs reversed defects in microglial maturation (178), suggesting early impacts of the maternal microbiome are temporary and/or reversible. Intraventricular injection of the SCFA propionic acid into adult rats activated microglia, promoted oxidative stress, and induced behavioral abnormalities (179), suggesting that excess access of SCFAs to the brain could be detrimental. Consistent with this, in a model of Parkinson’s disease, the altered microbiome contributed to disease symptoms and SCFA supplementation promoted α-synuclein–mediated neuroinflammation by activating microglia (180). As microglia play important roles in early neurodevelopment, additional research is warranted to dissect mechanisms by which the maternal microbiome affects microglial function in ways that impact the developing brain.

Many effector molecules of peripheral immune cells play fundamental roles in normal neurodevelopment. For example, various cytokines are present in the absence of overt inflammation and influence neurodevelopmental processes, such as neuronal proliferation, differentiation, migration, and synaptic plasticity (181). Recent research indicates that the maternal microbiome can modulate neurodevelopment by tuning cytokine responses to environmental challenges. In a mouse model of maternal stress, CCL2 and IL-6 were quickly elevated in the placenta and fetal brain in conventional, but not germ-free, dams (182). This microbiome-dependent regulation of brain CCL2 led to deficits in sociability and anxiety-like behavior in adult offspring. Similarly, maternal colonization with the Th17-inducing bacterium SFB enhanced IL-17A in the fetal brain, which contributed to fetal cortical defects and social behavioral abnormalities in the offspring (183, 184). These data indicate that the maternal microbiome can impact offspring neurodevelopment by regulating peripheral immune homeostasis and responses to environmental challenges.

## Future directions

The maternal microbiome, itself and through interactions with environmental risk factors, is emerging as an important modifier of long-term health trajectories for both mother and offspring. Many studies in animal models provide fundamental proof of concept for causality between the maternal gut microbiome and alterations in neurodevelopment and behavior in the offspring. Whether these principles faithfully translate to the human condition remains poorly understood. This is especially challenging given the concerns of and confounding issues related to conducting human studies with pregnant women and children as vulnerable groups. The wealth of evidence to date, drawn from laboratory animals and human epidemiological studies, supports the need for longitudinal evaluation of the maternal microbiome and related outcomes in maternal-offspring pairings. Results from these, together with advances in mechanistic studies in animal models, will form the foundation for further evaluating the promise of microbiome-based interventions for the perinatal period. Recent studies have begun to define the various pathways by which metabolites from, or modulated by, the maternal microbiome can impact the brain and behavior of the offspring. Much remains to be explored in terms of the molecular and cellular biology underlying the new phenomena that have been described, in addition to other non-metabolite-based pathways that may contribute. In addition, while the vast majority of studies on the maternal microbiome have focused on the maternal gut microbiome, additional research is warranted to evaluate the effects of microbiomes from other sites, such as the maternal vagina, milk, oral cavity, and skin, on the neurological health of the mother and offspring. Finally, a key area for future advancement requires acknowledging the importance of maternal health, in and of itself, rather than solely as a determinant of offspring health. The field is ripe for evaluating the microbiome-gut-brain axis in the context of women’s health, especially the maternal brain during pregnancy and the postpartum period. Understanding how maternal microbiomes impact both maternal and offspring brain health will not only uncover new knowledge regarding biological interactions that occur during important critical periods, but also pave the way for new approaches to addressing the unmet medical needs of women and children.

## Figures and Tables

**Table 2:**
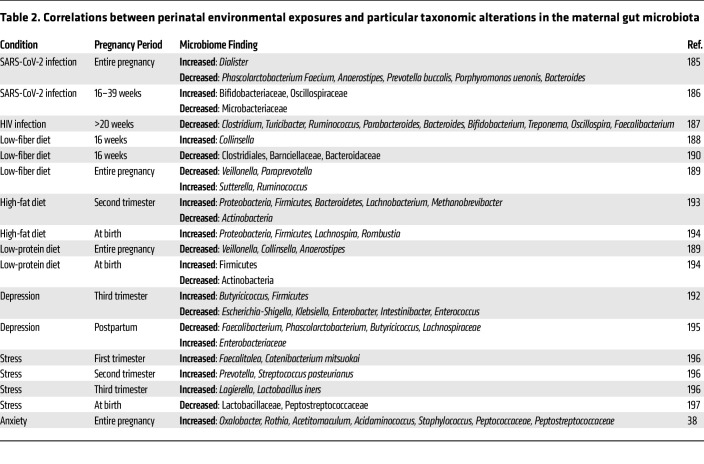
Correlations between perinatal environmental exposures and particular taxonomic alterations in the maternal gut microbiota

**Table 1:**
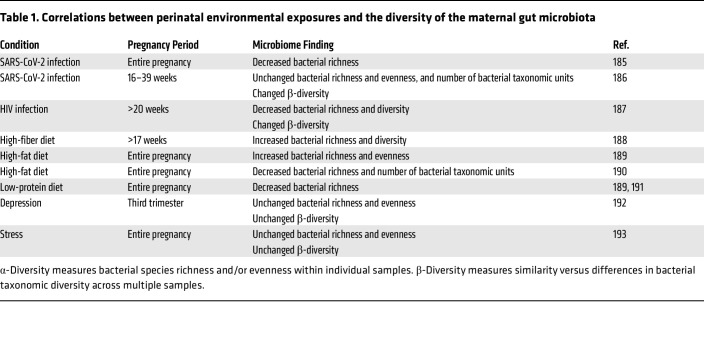
Correlations between perinatal environmental exposures and the diversity of the maternal gut microbiota

## Version 1 02/17/2025

Electronic publication
